# Changes of androgen and corticosterone metabolites excretion and conversion in cystic fibrosis

**DOI:** 10.3389/fendo.2023.1244127

**Published:** 2023-08-29

**Authors:** Rafał Podgórski, Marta Sumińska, Marta Rachel, Barbara Pikuła, Piotr Fichna, Martin Bidlingmaier, Marta Fichna

**Affiliations:** ^1^ Department of Biochemistry, Institute of Medical Sciences, Medical College of Rzeszow University, Rzeszow, Poland; ^2^ Department of Pediatric Diabetes, Auxology and Obesity, Poznan University of Medical Sciences, Poznan, Poland; ^3^ Department of Pediatric, Institute of Medical Sciences, Medical College of Rzeszow University, Rzeszow, Poland; ^4^ Department of Biology, Institute of Medical Sciences, Medical College of Rzeszow University, Rzeszow, Poland; ^5^ Endocrine Laboratory, Medizinische Klinik und Poliklinik IV, Klinikum der Universität München, Munich, Germany; ^6^ Department of Endocrinology, Metabolism and Internal Medicine, Poznan University of Medical Sciences, Poznan, Poland

**Keywords:** cystic fibrosis, androgen secretion, mineralocorticoid secretion, urinary steroid profile, adrenal insufficiency

## Abstract

Cystic fibrosis (CF) is a life-threatening inherited disease related to a mutation in the CFTR gene, that leads to serious health complications such as chronic pulmonary infections, pancreatic insufficiency, dysfunction of the sweat glands and reproductive system. For the first time, we have described the profile of corticosterone and androgen metabolites in urine, as well as the activity of enzymes involved in steroid genesis and metabolism in people with CF, using gas chromatography/mass spectrometry. A significant reduction in the excretion of most of the measured metabolites in CF was found. These differences were observed in the group of progestagen metabolites, as well as among metabolites of corticosterone and androgens. We revealed higher activities of 17β-hydroxysteroid dehydrogenase and 17,20-lyase in the Δ4 pathway compared with controls, what can promote the androgen synthesis through the backdoor androgen pathway. We have also found the increased conversion activity of 11-oxyganated steroids by 5a-reductase in backdoor pathway. Levels of the most potent and vital androgens (testosterone and dihydrotestosterone) are comparable in both groups. However, the excretion of dehydroepiandrosterone was lower in CF. Decreased cholesterol lipoprotein levels may contribute to limited intracellular cholesterol supply and reduced adrenal steroidogenesis in CF individuals. Changes in the activity of some steroidogenesis enzymes may suggest the presence of some peripheral adaptive mechanisms in CF to maintain androgen balance in the body despite the limited sufficiency of secretion by the adrenal cortex.

## 1 Introduction

Cystic fibrosis (CF) is one of the most frequent inherited autosomal recessive diseases. It is still an incurable systemic condition, currently affecting more than 100,000 people worldwide, with the highest prevalence in Caucasian populations. In the past, patients rarely lived to adulthood, but better treatment methods have now significantly increased the life expectancy of people with CF (1). The disease is caused by mutations in the cystic fibrosis transmembrane conductance regulator (*CFTR*) gene that lead to impairment of the function of the plasma membrane cyclic AMP-activated chloride and bicarbonate channels. These channels are highly expressed in several organs, such as the lungs, pancreas, kidney, intestine, vas deferens, and sweat ducts, where it is involved in the formation of a thin layer of mucosal fluid. Dysfunction of the CFTR channel causes mucus retention, what is the background for chronic pulmonary infection, and airway inflammation. Furthermore, the *CFTR* gene mutation also contributes to digestive system disturbances associated mainly with exocrine pancreatic insufficiency, biliary cirrhosis, sweat glands and reproductive system dysfunction (2, 3). Since 1989, when the *CFTR* gene was identified, thousands of mutations have been found, of which ΔF508 is the most frequent (4). *CFTR* expression is multifactorially regulated, and many mechanisms (including hormonal control) cause tissue-specific regulation induced by this gene. A recent study has found that estrogens can increase the presence of CFTR in the uterine epithelium of the oviductal mucosa (4, 5). Elements of the glucocorticoid response have been found in the CFTR promoter, and treatment with glucocorticoid changes the expression and activity of CFTR (6). Dexamethasone enhances the maturation and expression of the wild-type CFTR protein in the *in vitro* study, whereas the increase in fetal cortisol levels corresponds to the decrease in fetal CFTR expression (7, 8). However, little is still known about the effect of hormones on CFTR function. However, there is still little knowledge about the effects of hormones on CFTR function.

**Figure 1:**
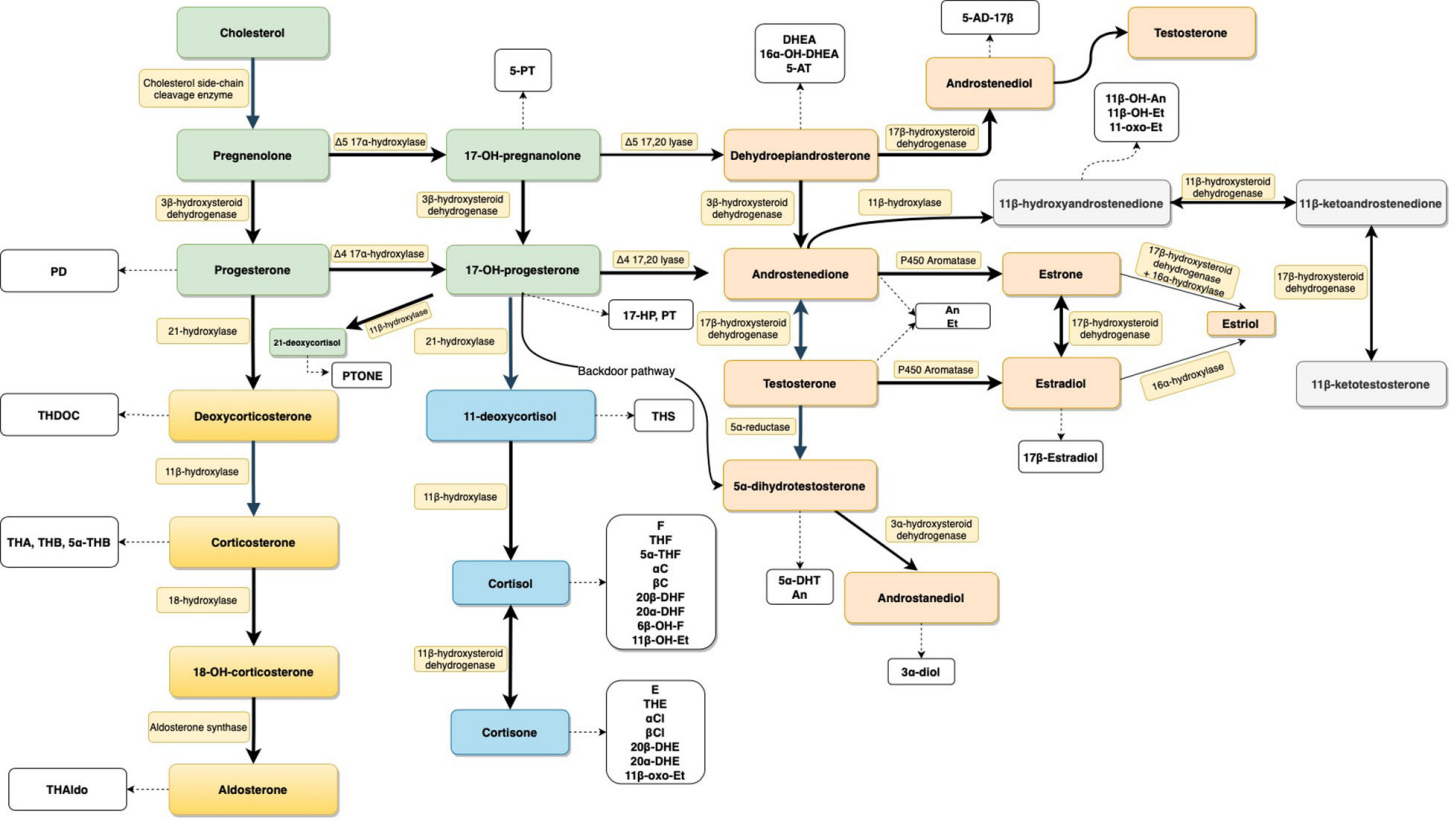
Schematic presentation of the steroid biosynthesis pathways. Steroids are colored according to their classes: general precursors (green), mineralocorticoids (yellow), glucocorticoids (blue), androgens (light orange), and 11-oxygenated androgens (light gray). Corresponding urinary metabolites are shown in white boxes. Enzymes involved in the pathway are represented in yellowish boxes. For metabolite abbreviations see **Table 1**.

Steroid hormones have an impact on the entire body and are engaged in various biological pathways, notably in the functioning of the reproductive system (androgens, estrogens, gestagens), as well as in the maintenance of metabolic homeostasis (glucocorticoids and mineralocorticoids). Steroid hormone biosynthesis (**Figure 1**) is a highly complex process regulated by numerous factors, such as the endocrine system (e.g. the mechanisms of negative feedback loop), the transcription and post-translational modification of steroidogenic enzymes, the ratio between free and bound circulating steroid compounds and peripheral metabolism (9, 10). Cystic fibrosis is found not to be directly associated with effects on adrenal glands, but alterations in adrenal hormone levels such as dehydroepiandrosterone (DHEA) have been reported (11). Reduced levels of DHEA are associated with chronic degenerative diseases related to aging, such as idiopathic pulmonary fibrosis (12).

**Table 1:** Urinary 24-h excretion rates of steroid metabolites in all subjects, boys and girls, respectively (Steroid excretion results were normalized per 1 m^2^ of the individual’s body surface area).

Steroid metabolite [μg/24h/m^2^]	All			Boys			Girls		
	CF	Control		CF	Control		CF	Control	
	Median (IQR)	Median (IQR)	p	Median (IQR)	Median (IQR)	p	Median (IQR)	Median (IQR)	p
Metabolites of early steps of steroidogenesis									
Pregnanediol (PD)	24.26 (9.28-50.35)	59.68 (30.96-115.93)	**0.001**	24.52 (8.87-40.57)	42.75 (26.45-82.72)	**0.047**	24.13 (9.28-79.3)	70.7 (45.38-199.6)	**0.006**
Pregnanetriol (PT)	58.67 (40.15-137.22)	165.33 (116.33-330.6)	**<0.001**	90.38 (44.5-151.78)	155.83 (96.08-286.21)	**0.030**	55.54 (28.93-128.93)	203.96 (129.59-407.18)	**<0.001**
17-Hydroxypregnanolone (17-HP)	4.14 (1.7-8.75)	7.97 (3.63-12.97)	**0.025**	6.11 (1.94-10.19)	7.38 (3.64-11.93)	0.448	2.84 (1.35-6.57)	8.23 (3.77-13.04)	**0.028**
Pregnanetriolon (PTONE)	2.11 (1.08-6.16)	6.87 (5.23-11.04)	**<0.001**	2.99 (1.94- 6.94)	6.98 (5.64-12.01)	**0.007**	1.39 (0.84-3.24)	6.87 (4.96-11.02)	**<0.001**
Pregnetriol (5-PT)	1.25 (0.62-4.14)	5 (2.53-15.91)	**<0.001**	2.26 (0.45-5.02)	5.36 (2.74-16.98)	**0.037**	1.08 (0.62-3.6)	3.83 (2.14-13.19)	**0.010**
Androgens and their metabolites									
Androsterone (An)	282.55 (118.71-638.47)	410 (182.48-1218.59)	0.083	357.72 (74.02-806.1)	308.31 (118.05-1218.59)	0.625	239.24 (120.93-426.98)	672.56 (278.62-1264.66)	**0.025**
Etiocholanolone (Et)	167.81 (62.74-429.79)	282.32 (110.45-595.29)	0.130	272.75 (37.41-451.76)	197 (70.53-592.39)	0.581	163.53 (69.06-198.22)	327.94 (136.14-783.16)	0.062
Androstanediol (3α-Diol)	10.17 (4.62-30.78)	18.35 (12.04-30.01)	0.206	22.94 (5.04-41.25)	21.04 (13.61-41.39)	0.747	9.47 (4.62-30.07)	15.51 (10.44-24.58)	0.225
Dehydroepiandrosterone (DHEA)	12.7 (4.53-49.64)	67.61 (15.2-243.31)	**<0.001**	19.76 (8.52-56.59)	126.72 (31.72-275.87)	**0.007**	6.37 (4.36-20.15)	19.43 (11.57-106.62)	**0.009**
Androstenediol (5-AD-17β)	5.95 (3.75-15.03)	9.99 (4.99-20.08)	0.367	6.78 (4.12-26.94)	8.75 (4.31-17.84)	0.843	5.95 (2.85-15.03)	12.83 (6.27-25.51)	0.234
11-oxo-Etiocholanolone (11-OXO-Et)	184.98 (137.24-325.62)	271.72 (169.05-393.27)	0.142	177.33 (138.61-94.29)	343.22 (224.77-395.85)	0.142	184.98 (135.65-269.74)	187.68 (143.14-383.01)	0.754
5α-Dihydrotestosterone (5α-DHT)	4.41 (2.34-9.47)	6.59 (4.09-11.67)	0.098	6.12 (2.37-10.86)	7.49 (4.27-13.11)	0.460	3.91 (2.25-7.36)	6.27 (3.87-10.95)	0.197
Testosterone (T)	11.26 (7.86-19.9)	9.42 (6.58-16.51)	0.493	11.01 (8.43-35.88)	9.82 (6.64-18.91)	0.313	11.26 (6.72-13.23)	9.02 (6.32-15.27)	0.793
11β-Hydroxyandrosterone (11β-OH-An)	257.49 (117.78-383.39)	464.17 (291.78-753.02)	**<0.001**	259.51 (110.71-582.88)	412.64 (271.95-628.67)	0.079	204.39 (117.83-302.1)	515.76 (327.94-841.49)	**<0.001**
11β-Hydroxyetiocholanolone (11β-OH-Et)	11.21 (7.28-17.85)	106.69 (48.63-203.96)	**<0.001**	11.81 (8.51-18.43)	133.66 (54.78-228.77)	**<0.001**	10.7 (6.8-17.85)	80.41 (34.6-191.08)	**<0.001**
16α-Hydroxy-DHEA (16α-OH-DHEA)	37.84 (20.67-141.98)	77.56 (38.73-190.58)	**0.016**	44.5 (23.51-145.82)	107.13 (46.26-201.45)	0.094	37.84 (12.84-62.32)	62.92 (30.32-173.71)	0.112
Androstenetriol (5-AT)	37.32 (13.38-173.13)	71.07 (34.82-148.83)	0.277	63.07 (15.81- 245.43)	71.08 (26.13-148.83)	0.992	31.72 (13.38-159.95)	72.47 (39.36-169.73)	0.150
Σ Androgens	836.06 (409.52-2025.86)	1438.65 (826.35-3221.96)	**0.012**	1038.43 (381.16-2466.50)	1372.45 (752.59-3086.47)	0.343	734.67 (493.53-1178.96)	1878.44 (881.41-3320.10)	**0.009**
Estrogens									
Estriol	0.41 (0.2-0.77)	0.85 (0.32-1.66)	**0.017**	0.36 (0.18- 0.53)	0.5 (0.24-1.11)	0.069	0.51 (0.28-1.14)	1.17 (0.68-2.8)	0.052
17β-Estradiol	0.45 (0.23-1.64)	0.44 (0.19-0.96)	0.326	0.41 (0.26- 0.71)	0.28 (0.15-0.73)	0.355	0.8 (0.21-2.14)	0.53 (0.24-1.11)	0.568
11-Deoxycortisol metabolite									
Tetrahydro-11-deoxycortisol (THS)	21.83 (8.58-29.4)	39.87 (26.5-63.03)	**<0.001**	23.57 (11.76-43.89)	39.67 (23.35-63.43)	**0.028**	15.58 (6.91-24.45)	42 (33.2-62.78)	**0.002**
Corticosterone metabolites									
Tetrahydrodeoxycorticosterone (THDOC)	1.35 (0.83-2.03)	3.46 (2.07-5.19)	**<0.001**	1.23 (0.87-2.48)	3.33 (2-4.46)	**0.003**	1.48 (0.55-1.76)	3.66 (2.42-5.33)	**0.001**
Tetrahydro-11-dehydrocorticosterone (THA)	47.86 (34.44-84.79)	84.99 (58.1-130.69)	**0.002**	55.3 (36.64-88.75)	82.89 (54.05-145.36)	0.052	38.1 (24.76-84.79)	90 (59.19-125.19)	**0.032**
Tetrahydrocorticosterone (THB)	28.71 (18.6-58.06)	52.67 (39.77-78.96)	**0.004**	43.25 (21.25-55.38)	47.36 (38.55-76.45)	0.154	23.3 (12.58-62.21)	57.33 (40.9-79.27)	**0.021**
5α-Tetrahydrocorticosterone (5α-THB)	112.26 (53.76-196.31)	199.57 (138.07-301.85)	**<0.001**	125.85 (76.86-227.7)	199.57 (133.39-301.85)	**0.033**	68.03 (49.54-128.28)	205.6 (143.51-290.71)	**0.001**
Σ Corticosterone metabolites	198.02 (95.9-281.62)	361.46 (251.53-519.36)	**<0.001**	254.43 (144.77-369.35)	352.14 (251.02-522.21)	**0.024**	163.32 (95.52-280.42)	375.87 (253.94-501.08)	**0.003**

IQR- interquartile range; Σ Androgens - the sum of: An, Et, 3α-Diol, DHEA, 5-AD-17β, 5α-DHT, T, 16α-OH-DHEA, 11β-OH-An, 5-AT; Σ Corticosterone metabolites - the sum of: THDOC, THA, THB, 5α-THB. Statistically significant values were bolded.

The analytical technique used in the study, gas chromatography-mass spectrometry (GC-MS), is a well-known and reliable method that is routinely applied to analyze the profiles of steroid metabolites in urine. Most steroidogenesis errors were first defined by urinary profiling, and it is still a highly valued diagnostic tool for the diagnosis of rare steroid disorders. Additionally, 24-hour urine collection carried out at home appears to provide a comprehensive insight into daily total steroid excretion, free from the influence of the stress effects, such as hospitalization, that can affect results (13–15).

This is the first study to describe the urinary profile of androgen and mineralocorticoid hormones and their metabolites, quantified by the GC-MS method, as well as the activity of the enzymes involved in the genesis and degradation of these steroids in patients with CF. The aim of this work is to gain knowledge of adrenal function and steroid metabolism in patients affected by CF.

## 2 Materials and methods

### 2.1 Subjects

The study was conducted on 25 affected persons (13 girls, 12 boys) and 74 healthy controls (32 girls, 42 boys) aged 5 to 18. Urine collection for 24 hours was carried out according to the standard protocol and was done at home to avoid additional stress associated with hospitalization. Parents and children were educated about the collection procedure and to ensure compliance, written instructions were given. The study protocol was approved by the Bioethical Committee of the University of Rzeszow (Decision number 3/12/2018) and all procedures were carried out in accordance with the Declaration of Helsinki. The diagnosis of CF was established on the basis of sweat chloride assay, genetic data, and immunoreactive trypsin test at neonatal age (patients born in 2009 or later). Further inclusion criteria for people with cystic fibrosis in the study were as follows: expiratory volume in the first second (FEV_1_) greater than 35% of predicted, stable pulmonary disease as defined by both clinical symptoms and no hospitalizations 30 days before screening. Exclusion criteria were: corticosteroid treatment, post-solid organ transplantation, liver and heart failure and psychiatric disorders.

All CF participants suffered from pancreatic insufficiency and were given pancreatic enzyme replacement therapy (Creon 25000, Solvay Pharmaceutical Inc., Marietta, Georgia, USA). Patients also received human DNase I recombinant (Pulmozyme, Genentech Inc., San Francisco, California, USA; one 2.5 mg ampoule inhaled once a day using a nebulizer), fat-soluble vitamins in the form of tablets ADEK (Scandipharm, Birmingham, Alabama, USA), supplemental nutritional drinks (Nutrison Protein Plus, Nutricia, Poland) and were inhaled with a solution of 3%-10% sodium chloride 3-4 times a day.

The healthy control group consists of volunteers of the same age and sex with no chronic disease or special diet in medical history or physical examination. Participants did not take any medication 30 days before the study. All participants were subjected to anthropometric measurements. Body mass index (BMI) was calculated according to routine formulas as kg/m^2^ and body surface area (BSA) as m^2^ using the Mosteller formula [square root of (height (cm) x weight (kg)/3600)].

### 2.2 Quantification of urinary steroid metabolites

24-hour urine samples were stored at ≤-20°C before assessing the steroid profile by the GC-MS method as described previously (16–19). Briefly, after fortification of 1.5 ml of urine sample with medroxyprogesterone (recovery standard), free and conjugated urinary steroids were pre-extracted on a Sep-Pak C18 column, conjugates were enzymatically hydrolyzed with sulfatase and β-glucuronidase/arylsulfatase and extracted once again on a Sep-Pak C18 cartridge. After the addition of known amounts of internal standards, methoxyamine-trimethylsily ether derivatives were formed. After purification on a Lipidex 5000 column, GC-MS analysis was performed by using a Shimadzu QP-2010 Ultra Plus gas chromatograph connected with a single-quadrupole Shimadzu QP-2010 Ultra mass spectrometer. The measured compounds were separated on a ZB-1ms column and detected in a selected ion monitoring mode.

**Supplementary File (Table S1)** provides detailed method-validation data for all quantified steroids.

### 2.3 Statistical analyses

All statistical calculations were done using Statistica 13 software (Dell Inc., Tulsa, OK, USA). Steroid excretion rates have been normalized to the body surface (BSA) due to the close anatomical and functional correlation between adrenal volume and BSA (20–22). The comparison between the studied groups was performed using nonparametric Mann-Whitney U tests, because most variables did not follow normal distributions. A p value below 0.05 was considered significant. Data are presented as median with interquartile range and/or mean ± SD.

## 3 Results

### 3.1 Baseline characteristics of the study population

25 patients affected by CF and 74 healthy volunteers were enrolled in the study. Mean values ± SD for basic anthropometric parameters, pulmonary function and clinical laboratory markers are shown in **Table 2**. The mean age, height, height Z-Score, weight, weight Z-Score BMI, BMI Z-Score as well as BSA were not significantly different between the participants. Biochemical measurements were performed only in the CF individuals during treatment, and attention is drawn to the low values that describe the lipid profile, which are close to the lower reference limits, even LDL cholesterol value can be classified as below this range.

**Table 2:** Baseline demographic and clinical data of the study participants.

	CF [mean ± SD]	Healthy controls [mean ± SD]	*p*
Sex (F/M)	13/12	32/42	
Age (years)	12.24 ± 3.62	10.74 ± 3.16	0.067
Height (cm)	151.88 ± 17.52	148.40 ± 19.54	0.416
Height Z-Score	-0.31 ± 0.98	0.11 ± 0.94	0.134
Weight (kg)	44.24 ± 15.70	40.74 ± 14.69	0.310
Weight Z-Score	0.20 ± 1.20	0.05 ± 0.92	0.355
BMI (kg/m^2^)	18.67 ± 3.55	17.83 ± 2.94	0.514
BMI Z-Score	-0.17 ± 0.97	-0.03 ± 1.03	0.355
BSA (m^2^)	1.35± 0.32	1.29 ± 0.31	0.292
Genotype			
Homozygous ΔF508. n (%)	20 (80.0%)	–	–
Heterozygous ΔF508. n (%)	5 (20.0)	–	–
Clinical laboratory markers			
CRP (mg/L)	5.54 ± 6.1	–	**- " **
IL-6 (pg/ml) Norm <7	6.19 ± 10.35		–
NEU (%)	61.01 ± 15.3	–	**-**
WBC (10^3^/µL)	9.95 ± 3.6	–	**-**
Cholesterol (mg/dL)	117.67± 17.43	–	–
HDL cholesterol (mg/dL)	42.89 ± 9.73	–	–
LDL cholesterol (mg/dL)	66.89 ± 16.53	–	–
Triglycerides (mg/dL)	84.7 ± 43.5	–	–
Pulmonary function			
FEV_1_ (L)	2.39± 0.87	–	**-**

BMI, body mass index; BSA, body surface area; IL-6, Interleukin 6; WBC, white blood cells; NEU, neutrophils; CRP, C-reactive protein; FEV1, forced expiratory volume in 1 second; data are presented as mean ± SD; differences between means were analyzed using Mann-Whitney U test.

### 3.2 Assessment of steroid excretion and enzyme activity

Quantitative analysis of 24 steroid metabolites in the urine was performed. The results of 24-hour urinary excretion rates of the major androgen and mineralocorticoid metabolites corrected for BSA are shown in **Table 1**. This table also provides a comparison of the daily renal output of steroid metabolites between study groups using the Mann-Whitney U test.

We observed characteristically lower urinary excretion of pregnane metabolites in patients with CF compared to healthy controls. Almost all differences were confirmed as statistically significant in both the analysis of all patients and separately in subgroups of girls and boys.

All mineralocorticoid metabolites had a significantly lower excretion rate in CF compared to the control and the largest decrease in median excretion of steroid metabolites was observed for THDOC (2.7 times lower in CF, p<0.001) and 5α-THB (1.8 times lower, p<0.001) and THS (2.0 times lower in CF, p<0.001).

Regard to the androgen metabolites, most have shown a statistically significant lower excretion in participants affected by CF (with the exception of An, Et, 3α-Diol and testosterone in boys). The most notable difference was indicated for 11β-OH-Et (9.8 times lower in CF, p<0.001) and DHEA (3.8 times lower in CF, p<0.001). It should be mentioned that the most potent androgens 5α-DHT and T had similar excretion rates.

We have also found a relevant decrease in estriol concentration in the urine of CF individuals. A significantly lower total content of the 10 main androgen metabolites (An, Et, 3α-Diol, DHEA, 5-AD-17β, 5α-DHT, T, 16α-OH-DHEA, 11β-OH-An, 5-AT; 1.7- fold, p<0.012) and 4 mineralocorticoid metabolites (THDOC, THA, THB, 5α-THB; 1.9-fold, <0.001) was also observed in the CF group.

The results were also separately evaluated for girls and boys affected by CF, and compared to their coevals from the healthy control. As shown in **Table 1** in general, the excretion of steroid metabolites was lower in boys affected by CF, and the largest difference was observed for 11β-OH-Et (11.3 times lower in CF, p<0.001), DHEA (5.5 times lower in CF, p<0.013), PTONE (2.3 times lower in CF, p<0.015) and THDOC (2.7 times lower in CF, p<0.017). Other metabolites are excreted at similar levels in the boys’ subgroup.

Excretion of steroid metabolites in girls affected by CF was also lower compared to healthy controls. It should be noted that these disparities are more pronounced in girls than in boys, and almost all metabolites show a statistically significant difference. The greatest variation in girls subgroup was found for 11β-OH-Et (7.5 times lower in CF, p<0.001), PTONE (4.9 times lower in CF, p<0.001), PT (3.7 times lower in CF, p<0.001), 5α-THB (3.0 times lower in CF, p=0.001) and THDOC (2.5 times lower in CF, p=0.001). The statistically significant differences were also for Σ corticosterone metabolites (2.3 times lower, p=0.003) and Σ Androgens (2.6 times lower, p=0.009).

The next step in data analysis was the evaluation of steroid enzyme activity by calculating the substrate/product conversion ratio of steroid metabolites, using formulas established previously in the literature, especially for the diagnosis of various forms of congenital adrenal hyperplasia (17, 19, 23, 24) and some data from our previous study (22). The ratios between CF individuals and controls were compared using the Mann-Whitney U test (**Table 3**). The substrate to product ratios of the steroid metabolite representing the activity of 21-hydroxylase were lower in CF, indicating an increase in the activity of 21-hydroxylase in this condition compared to the control. Similarly, in CF higher enzyme activity seems to be found for the 17β-hydroxysteroid dehydrogenase (17β-HSD) calculated by DHEA/5-AD-17β and 16α-OH-DHEA/5-AT ratio, but this finding was not statistically significant for girls. Evaluation of the Δ4-pathway activity of 17,20-lyase revealed a higher activity of this enzyme in CF but only two ((17HP+PT)/(An+Et) and PT/11ß-OH-An) of four calculated ratios were statistically significant. This observation was not confirmed when assessing Δ4 pathway activity for 17α-hydroxylase (17α-HSD). We have also found a predominance in the activity of the Δ4-pathway versus Δ5-pathway for 17α-hydroxylase/17,20-lyase (CYP17) enzyme complex in CF. Evaluation of 11β-hydroxylase activity is difficult due to equivocal results and appears to be lower for the Et/11β-OH-Et ratio in CF, but the other indicators used for the activity of this enzyme did not differ in the groups compared. The activity of 3β-hydroxysteroid dehydrogenase (3β-HSD), 17α-hydroxylase and 17,20-lyase, aromatase (CYP19A1), and P450 oxidoreductase did not differ between study groups. However, the activity of P450 oxidoreductase appeared to be lower in girls with CF. Statistical analysis also revealed a significant decrease in the activity of 5α-reductase (SRD5A) in CF, which is especially important in the context of the involvement of this enzyme in the metabolism of corticosteroids expressed by THB/5αTHB ratio and androgens T/5α-DHT.

**Table 3:** Steroid hormone enzyme activities calculated as substrate/product ratios and comparison between CF and healthy control in general population, boys and girls respectively.

21-hydroxylase activity									
Substrate/product ratio	CF	Control		CF (boys)	Control (boys)		CF (girls)	Control (girls)	
	Median (IQR)	Median (IQR)	p	Median (IQR)	Median (IQR)	p	Median (IQR)	Median (IQR)	p
100*PTONE/(THF+5αTHF+THE)	0.18 (0.09-0.27)	0.23 (0.18-0.32)	**0.011**	0.19 (0.11-0.34)	0.23 (0.18-0.32)	0.221	0.12 (0.08-0.23)	0.23 (0.17-0.3)	**0.023**
17HP/(THE+5αTHF+THF)	0.02 (0.001-0.003)	0.002 (0.001-0.004)	0.857	0.002 (0.001 -0.005)	0.002 (0.001 -0.004)	0.959	0.003 (0.001 -0.003)	0.003 (0.002 -0.004)	0.608
PT/(THF+5αTHF+THE)	0.04 (0.02-0.07)	0.06 (0.04-0.09)	0.053	0.04 (0.02 ± 0.07)	0.05 (0.03-0.07)	0.303	0.05 (0.03 - 0.07)	0.07 (0.05-0.11)	**0.037**
(17HP+PT+PTONE)/(THF+5αTHF+THE)	0.05 (0.03-0.08)	0.07 (0.05-0.1)	0.055	0.05 (0.03 ± 0.08)	0.06 (0.04-0.08)	0.303	0.05 (0.03 - 0.08)	0.08 (0.05-0.11)	**0.047**
3β-hydroxysteroid dehydrogenase activity									
5PT/(THF+5αTHF+THE)	0.002 (0.000-0.003)	0.002 (0.001-0.005)	0.260	0.002 (0.0003-0.003)	0.002 (0.001-0.005)	0.338	0.002 (0-0.002)	0.001 (0.001-0.005)	0.867
5PT/PTONE	0.71 (0.29-2.56)	0.68 (0.31-1.74)	0.978	0.92 (0.13- 3.53)	0.71 (0.44-2.23)	0.700	0.71 (0.3 - 2.56)	0.68 (0.27-1)	0.608
DHEA/(THF+5αTHF+THE)	0.01 (0-0.02)	0.02 (0-0.08)	0.069	0.02 (0-0.02)	0.04 (0.01-0.11)	**0.049**	0.01 (0.01 - 0.01)	0.01 (0-0.03)	0.930
11β-hydroxylase activity									
100*THS/(THF+5αTHF+THE)	1.29 (0.92-1.55)	1.29 (0.99-1.79)	0.407	1.07 (0.82-1.64)	1.29 (0.98-1.7)	0.486	1.35 (1.18 - 1.54)	1.41 (1.01-1.82)	0.573
An/11β-OH-An	1.19 (2.17-0.87)	0.97 (0.67-1.99)	0.478	1.21 (0.57-2.22)	0.79 (0.4-1.49)	0.423	1.19 (0.58 - 1.38)	1.21 (0.83-1.52)	0.890
Et/11β-OH-Et	16.73 (7.56-29.26)	2.79 (0.89-5.33)	**<0.001**	24.95 (6.33-35.83)	2.01 (0.72-4.65)	**0.001**	13.58 (7.54 - 23.33)	2.88 (1.42-12.93)	**0.011**
CYP17 global (17α-hydroxylase and 17,20-lyase) activity									
PD/(An+Et)	0.06 (0.04-0.11)	0.09 (0.05-0.15)	0.072	0.04 (0.02-0.17)	0.08 (0.05-0.15)	0.216	0.07 (0.06 - 0.1)	0.1 (0.06-0.14)	0.172
(THA+THB+5αTHB)/(An+Et)	0.36 (0.21-0.89)	0.5 (0.23-1.46)	0.795	0.36 (0.16-1.42)	0.63 (0.27-1.9)	0.526	0.36 (0.26 - 0.89)	0.28 (0.21-0.85)	0.491
17α-hydroxylase global activity									
(THA+THB+5αTHB)/(THF+5αTHF+THE)	0.11 (0.1-0.14)	0.12 (0.1-0.14)	0.769	0.12 (0.09 - 0.13)	0.12 (0.1-0.14)	0.610	0.11 (0.1 - 0.17)	0.12 (0.09-0.14)	0.310
17α-hydroxylase global Δ4-pathway activity									
PD/17HP	7.21 (4.06-11.54)	7.89 (4.7-15.94)	0.425	4.6 (3.22 - 10.88)	6.41 (3.43-12.8)	0.460	10.39 (7.14 - 15.15)	10.3 (6.74-19.92)	0.523
PD/PT	0.35 (0.2-0.53)	0.32 (0.21-0.48)	0.578	0.21 (0.19 - 0.44)	0.28 (0.18-0.43)	0.909	0.41 (0.33 - 0.57)	0.36 (0.26-0.59)	0.402
PD/(17HP+PT)	0.34 (0.19-0.49)	0.31 (0.2-0.47)	0.657	0.2 (0.17 - 0.41)	0.27 (0.18-0.41)	0.827	0.4 (0.28- 0.54)	0.35 (0.25-0.56)	0.460
17,20-lyase global activity									
(An+Et)/(THF+5αTHF+THE)	0.36 (0.14-0.54)	0.24 (0.09-0.5)	0.657	0.36 (0.1 - 0.65)	0.22 (0.07-0.44)	0.596	0.34 (0.14-0.49)	0.37 (0.17-0.52)	0.695
17,20-lyase Δ5-pathway activity									
5PT/(DHEA+16α-OH-DHEA)	0.03 (0.02-0.07)	0.04 (0.02-0.07)	0.416	0.03 (0.01 - 0.08)	0.04 (0.02-0.07)	0.827	0.03 (0.03-0.07)	0.05 (0.03-0.08)	0.229
17,20-lyase Δ4-pathway activity									
(17HP+PT)/(An+Et)	0.15 (0.11-0.27)	0.25 (0.18-0.39)	**0.002**	0.14 (0.12 - 0.34)	0.28 (0.17-0.5)	**0.047**	0.18 (0.11 - 0.26)	0.22 (0.2-0.29)	**0.047**
(17HP)/11β-OH-An	0.02 (0.01-0.02)	0.02 (0.01-0.02)	0.895	0.02 (0.02 - 0.02)	0.02 (0.01-0.02)	0.670	0.02 (0.01 - 0.02)	0.02 (0.01-0.02)	0.590
PT/11β-OH-An	0.34 (0.18-0.47)	0.38 (0.32-0.56)	**0.046**	0.28 (0.2 - 0.59)	0.36 (0.3-0.52)	0.388	0.39 (0.17 - 0.46)	0.42 (0.33-0.57)	**0.039**
(17HP+PT)/11β-OH-An	0.36 (0.2-0.5)	0.4 (0.32-0.57)	0.052	0.3 (0.21 - 0.6)	0.38 (0.31-0.54)	0.399	0.4 (0.19 - 0.49)	0.44 (0.35-0.58)	0.052
CYP17 total Δ4- vs. Δ5-pathway activity									
11β-OH-An/(DHEA+16α-OH-DHEA +5AD-17β)	3.11 (2.19-7.65)	3.07 (1.04-6.32)	0.256	3.07 (1.43 - 6.04)	1.41 (0.75-4.86)	0.160	4.16 (2.21 - 8.23)	4.49 (1.26-8.01)	0.970
11ßOH-An/5AD-17β	31.36 (18.06-42.94)	45.27 (28.71-73.96)	**0.008**	27.4 (17.87 - 54.63)	45.61 (29.1-82.18)	0.072	39.18 (18.46 - 41.04)	42.52 (28.7-63.45)	0.059
17β-hydroxysteroid dehydrogenase activity									
DHEA/5AD-17ß	1.81 (1.04-3.18)	7.12 (1.9-20.92)	**<0.001**	2.71 (1.12- 3.87)	14.05 (6.08-34.80)	**<0.001**	1.42 (0,79-2,24)	2.32 (1,08-5,71)	0,096
16α-OH-DHEA/5-AT	0.72 (0.5-1.23)	1.19 (0.82-1.98)	**0.026**	0.69 (0.49 - 1.08)	1.35 (1-2.11)	**0.006**	0.84 (0.61 - 1.69)	0.9 (0.6-1.39)	0.871
P 450 oxidoreductase activity									
PD/(THE+THF+5αTHF)	0.01 (0.01-0.03)	0.02 (0.01-0.03)	0.258	0.01 (0.01-0.03)	45.27 (28.71-73.96)	0.462	0.02 (0.01 - 0.03)	0.03 (0.02-0.05)	0.157
(17HP+PT)/(THE+THF+5αTHF)	0.05 (0.02-0.07)	0.06 (0.04-0.09)	0.064	0.05 (0.03-0.08)	0.07 (0.05-0.1)	0.338	0.05 (0.03 - 0.08)	0.07 (0.05-0.11)	**0.044**
Aromatase (CYP19A1)									
testosterone/17ß-estradiola	39.15 (8.35-59.49)	29.49 (12.35-86.07)	0.594	40.25 (12.4 - 87.83)	54.02 (13.53-116.11)	0.685	30.03 (6.38 - 48.22)	23.5 (9.62-39.3)	0.950
5α- reductase activity									
Et/An	0.62 (0.48-0.76)	0.59 (0.4-0.81)	0.807	0.68 (0.45-0.98)	0.06 (0.04-0.09)	0.665	0.59 (0.49 - 0.64)	0.52 (0.38-0.78)	0.990
11β-OH-Et/11β-OH-An	0.04 (0.03-0.07)	0.25 (0.12-0.45)	**<0.001**	0.03 (0.02 - 0.08)	0.28 (0.15-0.51)	**<0.001**	0.05 (0.04 - 0.07)	0.24 (0.06-0.36)	**0.001**
T/5αDHT	3.57 (1.03-5.8)	1.68 (1.07-2.78)	**0.035**	4.67 (1.5 - 7.33)	1.75 (1.06-2.78)	**0.011**	1.98 (0.91 - 4.07)	1.62 (1.13-2.74)	0.608
THB/5αTHB	0.34 (0.25-0.48)	0.24 (0.19-0.34)	**0.006**	0.33 (0.26 - 0.39)	0.25 (0.19-0.34)	0.098	0.41 (0.25 - 0.49)	0.24 (0.19-0.34)	**0.030**

Please refer to **Table 1**. Statistically significant values were bolded.

## 4 Discussion

Our study has shown for the first time that the levels of the major corticosterone and most androgen metabolites in patients with CF differ from healthy subjects and are significantly decreased in the CF group. Evaluation of the activity of enzymes involved in the steroidogenesis process did not reveal relevant changes in enzyme activities, except for 17β-hydroxysteroid dehydrogenase and Δ4-pathway activity of 17,20-lyase, which seems to show higher activity in CF. On the contrary, the activity of SRD5A was lower in mineralocorticoids degradation and conversion of testosterone to dihydrotestosterone, but higher in the metabolization of 11-oxygenated androgens. We have also found enhanced activity of Δ4-pathway compered to Δ5-pathway.

CF is a multisystemic disease that causes diffusion of multiple organs, particularly the lungs and pancreas. Impairment of pancreatic enzyme production occurs in 85% of CF Individuals (25). Patients with 2 severe CFTR variants tend to have an earlier onset of pancreatic insufficiency and a more severe course (26). Furthermore, the degree of pancreatic insufficiency is positively correlated with the risk of later development of cystic fibrosis-related diabetes (27). Insufficient pancreatic enzyme secretion results in malabsorption of nutrients and fat-soluble vitamins, which can be intensified by co-existing liver disease. At the same time, recurrent pulmonary infections can cause higher caloric requirements and reduced appetite. Dysfunction of these vital organs in CF must trigger many adaptation processes in the organism to limit the adverse effects of the disease. A significant role in this adaptive mechanism played the endocrine system, including the HPA axis, which acts *ad hoc* in stressful situations, as well as through long-term and rhythmic effects on metabolism in basic areas, including proteins, carbohydrates, and lipids.

Alterations in steroid metabolism have been described for several conditions affecting energy homeostasis, including anorexia nervosa, obesity, and thyroid disorders (28–30). As in our study, chronic fasting leading to significant reductions in urinary cortisol and androgen metabolites compared to healthy subjects has been reported in young adult women with anorexia nervosa, and these changes can be reversed with adequate nutrition (31). On the other hand, in disorders associated with an altered steroid profile, such as polycystic ovary syndrome (hyperandrogenism), weight loss reduces excess androgen (32). The mechanisms underlying the effects of malnutrition on steroidogenesis are not understood. Furthermore, several studies have reported changes in other enzymes involved in the regulation of appetite and nutrition in CF, such as leptin, ghrelin, or neuropeptide Y (33, 34).

Persistent respiratory inflammation and incidents of hypoxia, as well as nutritional disorders, may also modify the redox state of nicotinamide adenine dinucleotide (phosphates) (NADH/NAD^+^ or NADPH/NADP^+^), cofactors involved in many enzymatic reactions of oxidation/reduction, among others, glycolysis, the Krebs cycle, fatty acid oxidation, and the function of many steroid hormone biosynthesis enzymes, such as CYP17, 3βHSD, SRD5A or 17α-HSD and 17β-HSD (35–37).

Increased levels of oxidative stress markers were observed in plasma, serum, exhaled breath condensate and sputum from patients with CF, which can lead to structural modification of cellular proteins, and consequently to changes in their function (38, 39). Another factor that can affect steroid hormone synthesis is the altered profile of lipids in CF. Recent studies have described in CF individuals a lower level of low-density lipoprotein (LDL) and high-density lipoprotein (HDL) as well as total cholesterol, the substrate from which the steroidogenesis process begins and from which all steroid hormones are derived (40–42). It could be the result of pancreatic insufficiency and a disturbance in fat digestion and absorption. As in previous reports, the evaluation of the lipid profile in the CF study group shows a low and close to limit of the reference values for cholesterol lipoproteins and triglycerides, despite pancreatic enzyme replacement therapy. Particularly low levels of LDL, may limit intracellular cholesterol supply; necessary for the first step of steroidogenesis. Perhaps this situation affects the reduction of adrenal steroidogenesis in CF individuals (42).

The outcomes of steroid profile analysis in urine have shown a much lower excretion of almost all steroids whose production is restricted to the adrenal cortex. The study group had a wide age range; therefore, we normalized the results by relating them to the participant’s BSA. It has been proven that adrenal volume and BSA have a close functional and anatomical correlation and the 24-hour urinary excretion of free cortisol corresponds to the plasma concentration of free cortisol, and correction with BSA provided relatively constant cortisol values independent of age and Tanner’s stages of sexual development in a group of healthy children aged 2-17 years (43, 44).

The synthesis of steroid hormone precursors expressed by urinary concentrations of pregnanediol, pregnanetriol, 17-hydroxypregnanolone, pregnanetriolon and pregnetriol was significantly reduced in CF. These could be the result of a diminished adrenal response to ACTH stimulation or/and a reduced cholesterol supply. Furthermore, levels of corticosterone metabolites were significantly reduced. Our recent study has revealed a similar relationship with respect to glucocorticoid secretion in CF individuals (22). At the same time, the major androgen concentrations are on same level (5α-DHT) in both studied groups or even higher in CF (T), taking into account gender. An explanation for this may be that these most potent sex hormones are mainly formed outside the adrenal glands through peripheral transformation from DHEA or androstenedione and in the gonads. In addition, peripheral sex hormone metabolism and changes in enzyme activity may be other important factors in maintaining proper T and 5α-DHT levels. On the other hand, the most potent androgenic hormone produced in the adrenal cortex, DHEA, is secreted at almost 6-fold lower concentrations in CF compared to controls. DHEA philologically declines with age, but an exaggerated decrease has been associated with chronic degenerative diseases, as well as several diseases associated with aging, such as insulin resistance, systemic arterial hypertension, atherosclerosis, and Sjögren syndrome. The decrease in DHEA concentration is also related to pulmonary hypertension and cardiovascular diseases, immune abnormalities, osteoporosis, glucose intolerance, diabetes, and deterioration of lipid metabolism (12, 45, 46). The most important aspect in relation to CF is that DHEA shows antifibrotic properties, by inhibiting cell proliferation and inducing early cell cycle arrest by suppressing glucose-6-phosphate dehydrogenase activity in lung fibroblasts (47). Several reports confirm the reduced level of DHEA in the course of CF (11, 48, 49). DHEA has also been shown to decrease in states of malnutrition, such as anorexia nervosa (50). Another common CF complication is osteoporosis, which pathogenesis is not fully understood (51–53). Recent studies suggest that CFTR is expressed in human bone cells and may affect cell signaling pathways that may contribute to this abnormal pattern of bone turnover (54, 55). Ongoing inflammation can also lead to excessive bone resorption during acute exacerbations in CF (56). In addition, some researchers indicate the role of DHEA in the development of osteoporosis, including CF (57). Reduced plasma levels of DHEA have been observed in older women, as well as in adolescents with anorexia and low bone density, suggesting its anti-osteoporotic effect (58, 59).

Significant differences were also observed in the summary secretion of androgens (the sum of An, Et, 3α-Diol, DHEA, 5-AD-17β, 5α-DHT, T, 16α-OH-DHEA, 11β-OH-An, 5-AT), which was much lower in CF. CF is often associated with hypogonadism, especially in male patients. Androgens have a great impact on the function of the immune system, macule strength, and lung function (60, 61). The concentrations of the most potent androgenic hormones T and 5α-DHT were at levels similar to or even higher in the affected group compared to the control, similar to previous reports (62). On the other hand, contradictory results were also described (63, 64). These two hormones are produced mainly by the gonads or during peripheral conversion, so the impact of reduced adrenal activity is less evident and/or other factors may play a role, as impairment of liver function and changes in enzyme activities. As in previous reports, we found a significant decrease in the secretion of 11-oxysteroids as 11β-OH-An and 11β-OH-Et in CF, but unlike those studies, we did not observe a decline in 11-oxo-Et (65). 11β-OH-Et and 11-oxo-Et, compounds with 5β-configuration, are almost exclusively metabolites of cortisol and cortisone, while the 5α-compound, 11β-OH-An, largely are derived from the reduction of 11-β-OH-androstenedione and to a lesser extent from An. These findings may indicate the increased activity of 5α/5β ratio, which is supported by the increased activity of 5α-reductase, expressed by 11β-OH-Et/11β-OH-An ratio. Similar to the previous study, this difference between CF and controls was more pronounced in boys (65). However, the activity of SRD5A is the degradation of corticosterone metabolites (THB/5αTHB ratio) and conversion of testosterone to the most potent androgen dihydrotestosterone was lower. We can distinguish a few isozymes of 5α-reductase, from which the best known is 5α-reductase type 2 (SRD5A2) expressed in the testes, prostate, and genital skin (66). SRD5A2 is responsible for the conversion of T to 5α-DHT. The role of the second isozyme 5α-reductase type 1 (SRD5A1), which is expressed in the skin, brain, liver, and to a lesser extent in the prostate gland, is not fully clear. SRD5A1 is involved in the conversion of androstenedione to androsterone and probably for the degradation of circulating C21 steroids in the liver in preparation for urinary excretion (35). In CF, the nutritional status is poor and it is of special interest that the activity of SRD5A was also reduced in patients with anorexia nervosa and critical illness (53, 54). In contrast, liver function is often affected in CF, but the activity of SRD5A in nonalcoholic fatty liver disease was reported to be reduced (67).

It should be mentioned that differences in morbidity and mortality have been reported in the population of patients with CF around the world, with worse clinical outcomes in women than in men. Lung function in women deteriorates more rapidly than in men, and male patients survive an average of nine years longer than women (68). The origin of this “gender gap” is unclear and likely multifactorial, related to intrinsic physiological, biochemical, and nutritional differences between the sexes (69). Recent studies underline the role of sex hormones in contributing to differences in CFTR expression and function, airway mucus and fluid characteristics, and pulmonary infection and inflammation (70, 71). In general, androgens appear to exhibit anti-inflammatory effects, while estrogens pro-inflammatory action (61). Our study found differences in estriol level between study groups, but no differences were observed in the level of this hormone within the sex, nor in the levels of 17β-estradiol. Estrogens are involved in the conversion of *Pseudomonas aeruginosa* strains from nonmucoid to mucoid phenotypes in women with CF, that can be related to pulmonary inflammatory processes (72). Recent studies describe that estrogen increases mucus production in human airway epithelial cells, inhibits airway chloride secretion, leading to dehydration of the airway surface, and impaired mucociliary clearance (73, 74). Nonetheless, the direct impact of sex hormones on pulmonary function is still unknown.

We have also found a significant decrease in the excretion of corticosterone metabolites in CF. Mineralocorticoids, especially aldosterone, are necessary to regulate sodium reabsorption in the kidney, sweat glands, salivary glands, and colon (75). People with CF show an inability to retain salt through sweat, due to dysfunction of the CFTR channels in the sweat ducts, leading to an excessive concentration of Na^+^ and Cl^-^ (3 to 5 times higher normal) in sweat and finally serious and, at times, fatal complications through cardiovascular collapse, especially in high-temperature environment (76, 77). These changes in sweat composition are used for diagnostic purposes as the sweat test, which is the gold standard for the diagnosis of cystic fibrosis (78). CFTR-related salt loss through sweat glands, and consequently volume, activates the renin-angiotensin-aldosterone axis (RAA) and may lead to secondary hyperaldosteronism. Replenishment of electrolyte deficiencies normalizes the aldosterone level, the entire RAA axis, and corticosterone metabolites (79–81). No changes in aldosterone levels were also reported in CF (82, 83). Our results do not support this finding, as the excretion of corticosterone metabolites (THDOC, THA, THB, 5α-THB) was significantly lower in CF compared to controls, which could be explained by good disease control and management.

Our study indicated a higher 17β-hydroxysteroid dehydrogenase activity that could promote conversion in the backdoor pathway in CF compared to controls. Metabolites from the backdoor androgen pathway, such as An were at comparable levels in the study groups, with the exception of 11-hydroxymetabolites. These findings are supported by the increased activity of SRD5A in the conversion of 11-oxymetabolites, which was previously described (22). The backdoor pathway or alternative pathway is represented by a chain of enzymatic reactions through which 17-hydroxyprogesterone is converted to 5α-DHT without T as an intermediate (84). 17β-HSD is involved in the final step of all androgens and estrogens formation. This is a large group of the 14 isoenzymes that can function as reductases or oxidases of the 17-keto group, to convert inactive 17-ketosteroids into their active 17-hydroxy forms (66). 17β-HSD differ in tissue distribution, substrate specificity, and regulatory mechanisms, allowing each peripheral cell the necessary mechanisms to control intracellular androgen and/or estrogen levels (85). Similarly to our results, a higher 17-HSD activity of 17β-HSD was described in children with premature adrenarche, which is quite surprising, due to the fact that CF is often associated with delayed puberty (86, 87). The explanation could be that disruption of puberty onset in CF is related to disease severity and nutritional status, and proper patient management can counteract this process (88). Furthermore, in contrast to the control, the general cohort, as well as girls and boys with CF, had higher 17,20-lyase activity through the Δ4 pathway, likely leading to the backdoor pathway. 17,20-lyase is key for all sex steroid production and is the enzyme that comprises the second main activity of CYP17. CYP17 also has 17α-hydroxylase activity, which is essential to shift steroidogenesis beyond mineralocorticoid production (89).

We have also found the advantage of activity Δ4 over Δ5 in CF. The Δ4 and Δ5 pathway dynamics play an important role in adrenal disorders such as polycystic ovary syndrome, congenial adrenal hyperplasia, Addison’s disease, and hormone-dependent cancers (90, 91). Androsterone (An) is thought as metabolite characteristic for backdoor androgen pathway when etiocholanolone (Et) is rather close to classic (Δ5) or alternative (Δ4) androgen creation. Recent studies show that the point of conversion of pregnenolone to progesterone or 17-OH- pregnenolone, and even more so the point of conversion of 17-OH pregnenolone to DHEA or 17-OH-progesterone, represent branch points in the pathway where the steroid flux is diverted through the Δ4 or Δ5 branches of the pathway (92). In this context, increased 17,20-lyase activity probably shifts steroid production through Δ4 pathway. We also showed a higher activity of 21-hydrosylase in CF, especially in girls, which allows us to conclude that 21-hydroxylase deficiency could be clearly excluded in people with CF.

This is one of the few studies measuring the urinary steroid metabolome in patients affected by CF compared with age-matched controls. Nonetheless, there are some limitations to our study. This is a single-center study and the number of patients involved was low (N= 25). Furthermore, the age range of the participants was quite wide. Taking into account the large number of metabolites analyzed and the magnitude of observed differences in most of them, the study has high statistical power to detect differences between the study groups and shows robust results. However, it is important to be aware that the level of hormones in urine is the final depiction of their content, which reflects the average of many factors such as the secretory capability of the adrenal glands, the involvement of other glands releasing steroid hormones (gonads), the metabolic activity of organs (liver, kidneys, adrenal glands), the adipose tissue volume, the influence of pro-inflammatory cytokines or oxidative stress. For these reasons, it is difficult to clearly assess the activity of enzymes involved in steroid formation and metabolism.

## 5 Conclusions

In conclusion, a significant decrease in androgen and corticosterone metabolites excretion can be found in CF, as well as in the activity of enzymes involved in the formation and metabolism of steroids, probably caused by altered peripheral metabolism of steroids. The urinary steroid secretion pattern of CF suggests higher activities of 17,20-lyase in the Δ4 pathway and 17β-HSD compared with age-matched controls, that can promote androgen synthesis through alternate androgen pathways. The levels of almost all adrenal cortex products are lower, which can indicate adrenal dysfunction. On the other hand, the concentrations of the most potent and vital glucocorticoids (cortisol, cortisone) and androgens (T and 5αDHT) are comparable in both groups, which may suggest the development of some peripheral adaptive mechanisms in CF to counteract insufficient production in the adrenal glands (22). The clinical manifestations in CF, in addition to the respiratory system, are mainly expressed by dysfunction of the exocrine glands (pancreas, liver, sweat glands) which may be an indication that an underlying defect is operating in all cells of the body and that the defect may be quantitative and probably regulatory in nature. Changes in steroid hormone metabolism may be another important factor associated with CF and should be taken into account when planning effective and comprehensive therapy. For example, it could be checked if the administration of DHEA may improve respiratory as well as immune system function and bone mineral density in CF. However, the conclusions of the paper should be regarded mainly as hypotheses that require confirmation in further studies.

## Data availability statement

The original contributions presented in the study are included in the article/**Supplementary Material**. Further inquiries can be directed to the corresponding author.

## Ethics statement

The studies involving humans were approved by Bioethical Committee of the University of Rzeszow. The studies were conducted in accordance with the local legislation and institutional requirements. Written informed consent for participation in this study was provided by the participants’ legal guardians/next of kin.

## Author contributions

Conceptualization, RP, MR, MS and PF. Methodology, RP, BP. Validation, MF and MB. Formal analysis, PF and MS. Investigation, RP. Resources, RP. Data curation, RP. Writing—original draft preparation, RP. Writing—review and editing, MS and PF. Supervision, MB. All authors have read and agreed to the published version of the manuscript.

## Supplementary material

Supplementary File 1 Method validation.
