# Supplementary material for: Changes of androgen and corticosterone metabolites excretion and conversion in cystic fibrosis
Source: Front Endocrinol (Lausanne). 2023 Aug 29;14:1244127. doi: 10.3389/fendo.2023.1244127 (PMC10497873; doi:10.3389/fendo.2023.1244127)
Supplement: Supplementary File 1 — Method validation. [file DataSheet_1.pdf]

Table S1. List of steroid compounds measured in urine.

Trivial name	Abbreviation	Systematic name	M	RT	QIon	Ref. Ion	LOD	LOQ	R^2	CV1	CV2	Rec
Androsterone	ANDRO	5α-androstan-3α-ol-17-one	290.4	13.5	270.2	360.2	229	693	0.9999	5.8	6,5	101
Etiocholanolone	ETIO	5β-androstan-3α-ol-17-one	290.4	13.7	270.2	360.2	135	410	0.9998	5.6	3,7	97
Androstanediol	3α-Diol	5α-androstan-3α, 17β-diol	292.4	13.7	331.3	241.2	46	139	0.9998	8.9	5,8	89
Dehydroepiandrosterone	DHEA	5-androsten-3β-ol-17-one	288.4	14.4	268.2	358.2	169	512	0.9998	8.8	7,7	81
Androstenediol	5-AD-17ß	5-androsten-3β, 17β-diol	290.4	14.8	239.2	344.2	106	322	0.9998	6.7	6,2	73
11-oxo-Etiocholanolone	11-OXO-ETIO	5β-androstan-3α-ol-11, 17-dione	304.4	15.1	269.2	405.3	69	208	0.9992	2.9	7,5	94
5α-Dihydrotestosterone	5α-DHT	5α-androstan-17β-ol-3-one	290.4	15.2	391.3	360.2	238	720	0.9994	4.6	6,4	98
17ß-Estradiol	17β-Estradiol	1, 3, 5(10)-estratrien-3, 17β-diol	272.4	15.4	416.4	285.1	16	47	0.9996	7.9	4,3	88
Testosterone	T	4-androsten-17β-ol-3-one	288.4	15.6	389.3	268.2	80	242	0.9991	3.4	6,6	125
11ß-Hydroxyandrosterone	11β-OH-An	5α-androstan-3α, 11β-diol-17-one	306.4	16.4	268.2	448.4	172	521	0.9998	9.0	7,8	111
11ß-Hydroxyetiocholanolone	11β-OH-Et	5β-androstan-3α, 11β-diol-17-one	306.4	16.7	268.2	448.4	114	346	0.9998	4.5	5,2	108
17-Hydroxypregnanolone	17-HP	5β-pregnan-3β, 17-diol-20-one	334.5	17.0	476.4	364.2	136	412	0.9998	10.0	9,0	84
16α-Hydroxy-DHEA	16α-OH-DHEA	5-androsten-3β, 16α-diol-17-one	304.4	17.1	266.2	446.3	86	262	0.9999	3.6	1,2	96
Pregnanediol	PD	5β-pregnan-3α, 20α-diol	316.5	17.7	269.2	284.2	41	125	0.9996	8.6	5,5	84
Pregnanetriol	PT	5β-pregnan-3α, 17, 20α-triol	336.5	18.3	435.4	255.2	105	318	0.9996	4.4	4,1	91
Androstenetriol	5-AT	5-androsten-3β, 16α, 17β-triol	306.4	19.3	432.4	522.4	75	228	0.9997	2.8	5,3	90
Tetrahydro-11-deoxycortisol	THS	5β-pregnan-3α, 17, 21-triol-20-one	350.5	19.6	564.5	474.4	39	120	0.9996	4.0	9,2	99
Tetrahydrodeoxycorticosterone	THDOC	5β-pregnan-3α, 21-diol-20-one	334.5	19.9	476.4	507.4	26	78	0.9990	5.6	2,7	86
Estriol	Estriol	1, 3, 5(10)-estratrien-3, 16α, 17β-triol	288.4	19.9	504.4	311.2	22	67	0.9994	1.9	2,9	96
Pregnanetriolon	PTONE	5β-pregnan-3α, 17, 20α-triol-11-one	350.5	20.6	449.4	359.2	54	163	0.9992	1.7	3,5	108
Pregnetriol	5-PT	5-pregnen-3β, 17, 20α-triol	334.5	21.6	433.4	343.3	15	45	0.9999	3.5	4,6	34
Tetrahydro-11dehydrocorticosterone	THA	5β-pregnan-3α, 21-diol-11, 20-dione	348.5	22.2	490.4	474.4	461	1397	0.9994	1.8	3,1	97
Tetrahydrocorticosterone	THB	5β-pregnan-3α, 11β, 21-triol-20-one	350.5	22.7	564.5	474.4	19	57	0.9998	4.9	2,6	99
5α-Tetrahydrocorticosterone	5α-THB	5α-pregnan-3α, 11β, 21-triol-20-one	350.5	23.1	564.5	472.3	2065	6257	0.9997	4.9	5,4	107

Abbreviations list: M-molar mass [g/mol], RT- Retention time [min], QIon- QIon: quantifier ion [m/z], Ref. Ion- Reference Ion [m/z] RT: retention time [min]. LOD: limit of detection [pg per sample]. LOQ: limits of quantitation [pg per sample]. R^2: correlation coefficient of the linear calibration

curve Rec: Recovery in %. CV1: intraassay coefficient of variation [%] (n = 6). CV2: interassay coefficient of variation [%] (n = 6). CV1 and CV2 were determined for a urine volume of 1.5 mL. OH: hydroxy, DH: dihydro, TH: tetrahydro.
